# Clinical Effects of Gamma-Radiation-Resistant* Aspergillus sydowii* on Germ-Free Mice Immunologically Prone to Inflammatory Bowel Disease

**DOI:** 10.1155/2016/5748745

**Published:** 2016-08-18

**Authors:** Alexander Rodriguez-Palacios, Natalia Aladyshkina, Mauricio Retuerto, Christopher L. Hager, Sanja Ilic, Mahmoud A. Ghannoum, Fabio Cominelli

**Affiliations:** ^1^Division of Gastroenterology and Liver Diseases, Department of Medicine, Digestive Health Research Institute, Case Western Reserve University School of Medicine, Cleveland, OH 44106, USA; ^2^Cleveland Digestive Diseases Research Core Center, Case Western Reserve University School of Medicine, Cleveland, OH 44106, USA; ^3^Center for Medical Mycology, Department of Dermatology, Case Western Reserve University School of Medicine, Cleveland, OH 44106, USA; ^4^Department of Human Sciences, Human Nutrition, College of Education and Human Ecology, The Ohio State University, Columbus, OH 43210, USA; ^5^Digestive Health Institute, University Hospitals Case Medical Center, Cleveland, OH 44106, USA

## Abstract

We report and investigated a case of inadvertent contamination of 125 mice (housed in two germ-free positive-pressurized isolators) with emerging human and coral pathogen* Aspergillus sydowii*. The infected mice correspond to genetic line SAMP1/YitFc, which have 100% immune predisposition to develop Crohn's disease-like spontaneous pathologies, namely, inflammatory bowel disease (IBD). Pathogen update based on a scoping review of the literature and our clinical observations and experimentation are discussed. The unwanted infection of germ-free mice (immunologically prone to suffer chronic inflammation) with human pathogen* A. sydowii* resulted in no overt signs of clinical disease over 3-week exposure period, or during DSS-induced colitis experiments. Results and observations suggest that* A. sydowii* alone has limited clinical effect in immunocompromised germ-free mice or that other commensal microbial flora is required for* Aspergillus*-associated disease to occur.

## 1. Introduction 

To date, there are a few studies reporting the severity of primary and disseminated gastrointestinal aspergillosis in immunocompromised hosts, including children [1] (see also references in [2]). Although aspergilloses are primarily confined to aerobic surfaces, systemic infections and rapid mortality are known to have occurred in humans and domestic animals [3], especially during disruption of the gastrointestinal barrier and associated microbiota (dysbiosis), which increases susceptibility to fatal systemic fungal infections [2]. Of all invasive fungal infections, aspergilloses account for 24% of human patients. With aspergillosis, one in every five patients develops fatal invasive infection.

Of epidemiological interest, we report a well-known fungal pathogen of marine corals and humans,* Aspergillus sydowii*, as an unwanted contaminant of a germ-free (GF) colony of SAMP/YitFc mice immunologically prone to suffer spontaneous inflammatory bowel disease (IBD; Crohn's-like intestinal disease) and other multiorgan inflammatory complications. Such Crohn's-like intestinal disease occurs in 100% mice in both specific pathogen-free (SPF) and germ-free conditions. Among the aspergilloses [3],* A. sydowii* is epidemiologically interesting because it is a pathogen known for causing mortality in Sea Fan corals (*Gorgonia ventalina*; animals that feed on plankton), threatening various Caribbean reefs since the 1990s [4], where it seems to remain geographically confined. Infections cause circular purple areas of necrosis and mortality that is not evidenced in other corals. Initially isolated from humans in the USA in 1989 from integumentary (e.g., skin and nails) infections [5],* A. sydowii* has been isolated in recent years from patients in other more distant regions (Czech Republic, Russia) [6, 7].

Mice under germ-free conditions have a very high risk of exuberant infections by any commensal, pathogenic, or environmental microbes. Here we report a case of 125 germ-free SAMP/YitFc mice inadvertently exposed to* A. sydowii* via commercial germ-free grade irradiated contaminated feed. The (SAMP/YitFc) mice contaminated in this study were particularly deemed prone to* A. sydowii* opportunistic fungal infections because (i) they were germ-free and therefore lacked protective competitive surface microbial commensal flora (microbiota), in both gut and skin; (ii) the mouse genetic line is immunologically prone to progressive chronic intestinal inflammation, conjunctivitis, and ulcerative dermatitis; (iii) they have decrease clearance of pathogens like* Salmonella enteritidis* [8]; and (iv) because germ-free mice have no protective antibody-mediated adaptive (memory) immunity against microorganisms due to lack of exposure to commensal or pathogenic microbes when raised in germ-free conditions.

## 2. Case 

**Figure 1:**
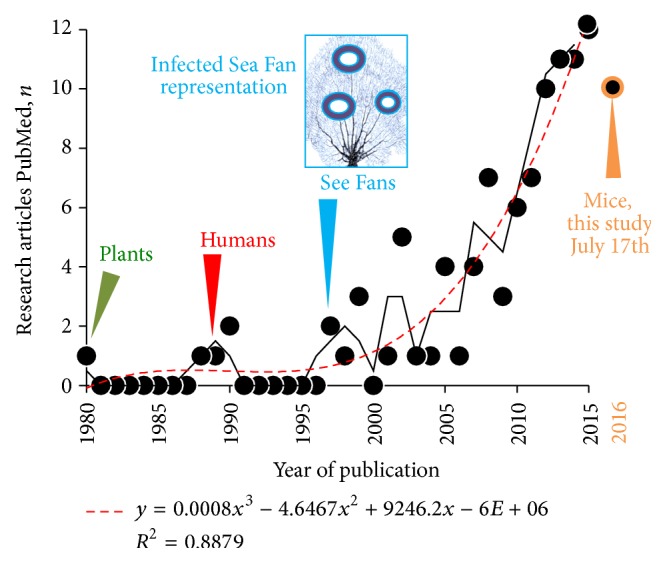
The frequency of peer-review studies on* Aspergillus sydowii* has increased exponentially over the last decade, with the curve shoulder starting after the fungus was linked to emerging epidemic infections in Caribbean corals. The first report was in plants in Egypt, 1980 [9]. Systematic scoping review of articles in PubMed until February 2016. Two-step moving average (solid line) and polynomial estimation curve (dashed line; *y* = *n* of publications estimate; *x*, year).

To historically contextualize the relevance of the present report, we first conducted a systematic scoping review of publications on* A. sydowii* since its discovery in plants in 1980 [9] until February 15, 2016, using PubMed and “*Aspergillus sydowii*” as search keyword term. We then examined the 100 top hits in Google Scholar (i.e., articles with the highest citations) and secondarily reviewed the title relevance of their Google citations referencing the papers for the years 2013–2015 to capture recently published studies with low citation indexes. Analysis of titles, abstracts, and content of 97 selected research studies indicates that the frequency of studies on emerging bioengineering and basic research on* A. sydowii* is growing exponentially since its isolation from affected Sea Fan corals was linked to the near extinction of some reefs in the West Indies in the 1990s (Figure 1). With the scoping review it also became evident that little is known about the intestinal colonization and pathogenicity of* A. sydowii* in mammals. Because the putative role of the mycobiome as disease contributor in human inflammatory bowel disease (IBD) is increasing, we deemed pertinent to report measurable mortality and clinical morbidity outcomes during this contamination event to further understand the role of the mycobiome in animals. Of relevance to IBD, the affected genetic mouse line is one of only two available spontaneous mouse models for Crohn's disease-like enteritis, primarily affected by ileitis (inflammation of ileum, i.e., the last segment of the small intestine).

Fungal infections are opportunistic and commonly occur in immunocompromised hosts [4, 6], in both humans and animals. Of immunological relevance, the SAMP1/YitFc mice correspond to a genetic line known for suffering spontaneous progressive intestinal inflammation, with unique three-dimensional structural similarity to human Crohn's disease lesions [10]. SAMP1/YitFc mice also have complex immunological abnormalities with 100% penetrance. Although partially understood, the immunological abnormality in these mice appears to transition from T-helper-1 (Th1) driven inflammation at young age to an inflammatory process driven by a combined Th1/Th2 alteration in animal adulthood which accentuates as disease worsens. Mechanistically, mice have gut epithelial barrier and pathogen clearance dysfunctions [8, 11], which could enhance their predisposition in GF conditions to overt fungal infections.

**Figure 2:**
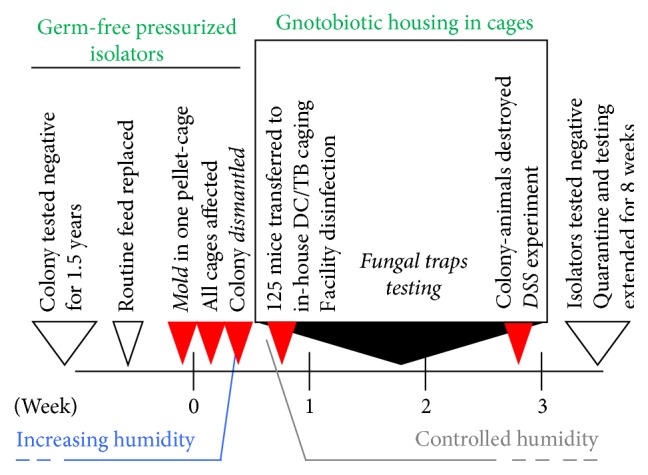
Timeline of* A. sydowii* contamination of germ-free mouse facility and implementation of germ-free in-house cage-based housing system to monitor 125 animals and conduct DSS colitis experimentation.

Herein, we describe the unexpected rapid and simultaneous contamination of 125 mice housed in two GF positive-pressurized isolators with* A. sydowii* in an established GF breeding facility (Cleveland, OH, USA) in June of 2015. An overview of the timeline during the contamination event and subsequent experimentation is presented in Figure 2 (i.e., fungus detection, colony dismantling, and monitoring mice for signs of disease using an in-house caging system).

In brief, two GF isolators, having weekly husbandry and high mouse density (2-3 breeders/cage; <10 unweaned mice/cage; 12–14 cages/isolator), were observed at maintenance to have unexpected condensation and increased humidity as well as containing feed pellets covered with mold on a day of routine inspection in June 2015 (day 0). Following removal, surface disinfection with Spor-Klenz® (1% hydrogen peroxide, 10% acetic, and 0.08% paracetic acid; Steris Corp., Catalog 6525) and quarantine of all cages (no handling/opening of lids) was initiated. Visual inspection of feed and bedding material revealed that most cages in both isolators exhibited various degrees of contamination within a week of mold detection. Due to the severity, extent, and the rate of feed contamination, the colony was dismantled, and tests for fungal identification were performed. All mice were transferred to an in-house cage system, and each cage and animal were monitored for overt signs of depression, diarrhea, and ocular, skin, or respiratory disease at the population level (using available scoring systems if needed) to determine the role of the infection in the overall health status of the mice. All mice were inspected daily maintaining germ-free grade sterility, to prevent contamination by other microbes.

**Figure 3:**
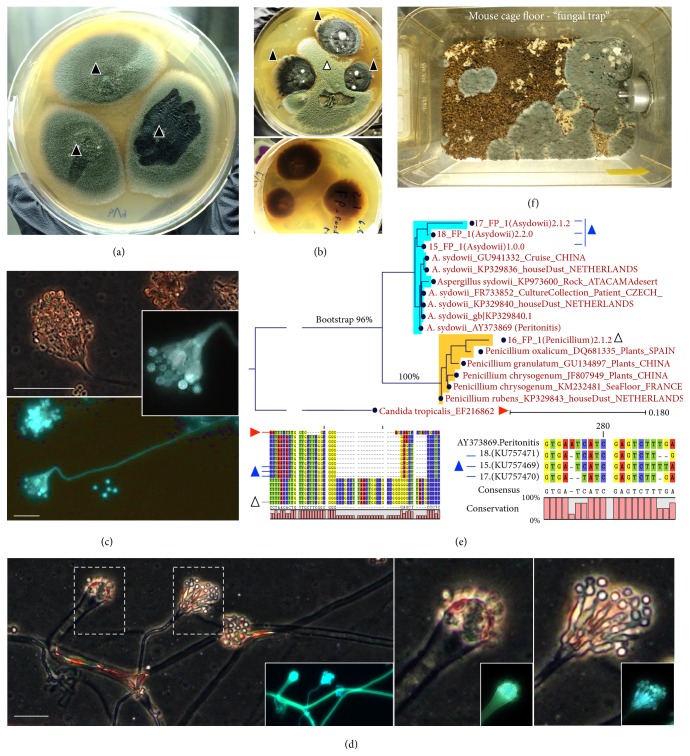
Culture and molecular identification of* Aspergillus sydowii*. (a)* A. sydowii* in potato dextrose agar supplemented with gentamicin and chloramphenicol (30 and 125 mg/L) with typical blue-green colonies (solid triangles, three distinct isolates from GF-irradiated rodent feed in two GF isolators). (b) Coculture with a* Penicillium* feed isolate (open triangle) to emphasize colony morphology and pigment production by* A. sydowii* (transagar pigmentation). (c) Adhesive tape preparation of* A. sydowii* colony to illustrate conidiophores, conidia, and typical fruiting head vesicle arrangement with nonseptate conidiophore (400x; phase-contrast microscopy on Calcofluor White from Remel; Alexa Fluor 430; emission 541; absorption 433 nm; scale bar, 30 *μ*m). (d) See distinct types of conidiophore formation by* A. sydowii*. (e) Neighbor-Joining phylogram of* A. sydowii* isolates in the context of other published 18S internal transcribed spacers ITS1–4 sequences and* Penicillium* spp. Notice the close-up of region where strains have sequence dissimilarity. (f) Panoramic photo of mouse cage bedding (set as “fungal trap”; i.e., mouse cage incubated for 3 weeks at 23°C) at the end of clinical observation period confirming mice were still exposed or shedding* A. sydowii*.

Over the following 2-week period the mold was confirmed as* A. sydowii*, and the animals were determined to be clinically sound, indicating that heavy exposure to* A. sydowii* spores do not necessarily result in mortality, or overtly respiratory, ocular, dermatological infections, or diarrhea in GF mice prone to immune deficiencies and intestinal disease. Single colony PCR and Sanger sequencing of 18S internal transcribed spacers (ITS1–4) indicated that three fungal isolates from both isolators were highly similar to* A. sydowii* sequences deposited in GenBank (Figures 3(a)–3(e)). Primers ITS1 (5′TCC GTA GGT GAA CCT GCG G) and ITS4 (5′TCC TCC GCT TAT TGA TAT GC) were used in a PCR amplification protocol described in detail in [12], using Dream Taq Green PCR Master Mix (Thermoscientific), 0.1 g/L bovine serum albumin, 1% of dimethyl sulfoxide, 6 mM MgCl_2_, and a final primer concentration of 400 nM. Initial denaturation at 94°C for 3 min was followed by 35 cycles of denaturation for 30 s each at 94°C, annealing at 50°C for 30 s, and extension at 72°C for 1 min. Upon cycling completion, there was a final extension time of 5 min at 72°C. PCR amplification of the ribosomal internal transcribed spacer 1 (ITS-1) and ITS-2 regions has been recommended as targets for molecular identification of medically important* Aspergillus *and as deposition of sequences at GenBank [13]. The ITS region 4 was also sequenced in this study; sequences from this report are available in GenBank under accession numbers KU757469, KU757470, and KU757471. “Fungal traps” (soiled moistened mouse cages incubated for 3 weeks at 23°C) with cages used during the clinical observation period confirmed the presence of* A. sydowii* (Figure 3(f)), supporting the lack of disease induction in these immune-defective inflammation-prone mice despite the presence of mold in the feces/bedding material.

**Figure 4:**
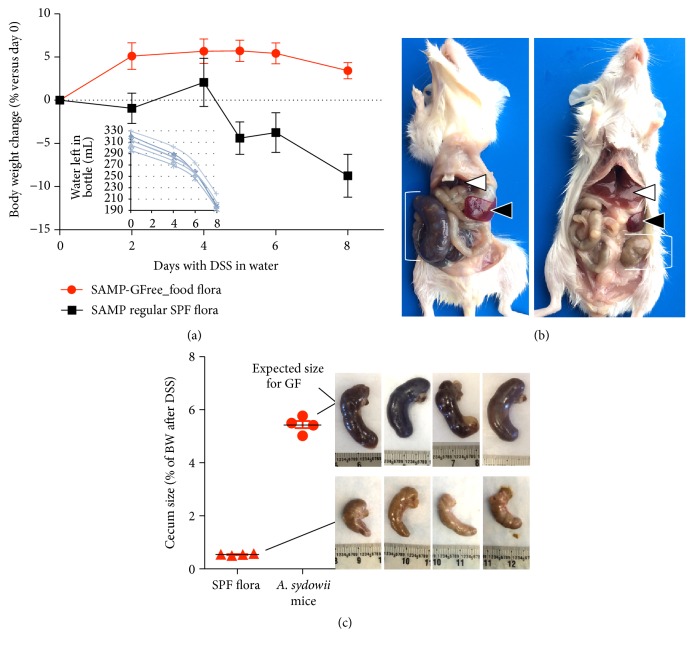
DSS treatment did not induce typical signs of DSS colitis in contaminated mice. (a) Body weight of mice (8 weeks old) contaminated with* A. sydowii* did not lose weight compared to mice harboring regular mouse SPF microbiota (*n* = 4/group, 2/cage). Inset plot: water content in each cage water bottle indicates that all mice consumed the same amount of DSS supplemented water. (b) Postmortem after DSS experiment. Note the small liver and large spleen and cecum (left) in* A. sydowii* mice as it is expected in mice in GF conditions. (c) Note the expected reduction in cecum size in SPF mice after DSS treatment due to diarrhea and appetite loss.

Given the reported risk for integumentary infections in humans, the rapid growth of the mold, and the critical need to eradicate it from the GF equipment and facility, all animals were euthanized after the clinical observation period, leaving a small cohort of mice for BSL-2 grade experimentation to determine the role of the fungal infection on the severity of acute colitis induction with dextran sulfate sodium (DSS). DSS colitis is a valid established method to assess the role of the gut microbiome in infection pathogenicity, including* Candida* spp., and the immunology of inflammatory bowel diseases [10, 14]. In brief, 7 days of dextran sulfate sodium supplementation (DSS) in the drinking water (3%, w/v) leads to the colitis in the presence of gut microbiota. Compared to SAMP/YitFc mice colonized with regular specific pathogen-free gut microbiota, the GF mice contaminated in this study did not exhibit clinical signs of acute colitis based on clinical signs that are typical for DSS-induced diarrhea, body weight loss, or reduction of the cecum size (Figures 4(a)–4(c)).

After the dismantling of the isolators and the euthanasia of the entire GF mouse colony, stringent disinfection protocols have been implemented. These were based on quaternary ammonium-based cleaner to remove organic matter, 70% ethanol to remove grease and dehydrate, and Spor-Klenz to eliminate microbial spores (bacterial and fungal) on delicate GF equipment. Floors and other surfaces were disinfected with Spor-Klenz and Clidox® (chlorine dioxide, Pharmacal, Catalog 96120F). Contaminated cages were handled and animals manipulated (e.g., for fecal sample collection and body weight measurements) exclusively in biosafety hoods equipped with new high-efficiency particulate air (HEPA) filters, routinely sterilized with chlorine gas. By July 2016, a year after the contamination event, the GF colony has been reestablished with amended protocols for autoclaving nutritionally balanced irradiated diet to increase feed safety. No further contamination events with this mold have been observed.

## 3. Discussion 

This is the first report of a contamination event in germ-free animals with increased susceptibility to inflammatory bowel disease by any fungal pathogen. We report* A. sydowii* feed-mediated exposure for the first time in GF animals devoid of natural protective commensal (intestinal and cutaneous) microbiota, in a genetic background with increased susceptibility to microbial infections. In humans, this pathogen can cause peritonitis [15], but no intestinal diseases have been reported for this microorganism. Although the intestinal passage of* A. sydowii* in snails (vectors for Sea Fan disease) results in excretion of viable spores [16], we did not detect their presence in mouse feces using culture agar plates, indicating that intestinal colonization was negligible or that, alternatively, the intestinal transit and/or metabolites could make spores superdormant, a phenomenon known to occur by other means in other spore-forming microorganisms, namely,* Bacillus *spp. and* Clostridium difficile* [17]. Molecular detection methods [14] were not employed to detect fungal spores in feces, but (i) the presence of* A. sydowii* at the end of the observation period in the mouse cages and fecal excrements using fungal traps, (ii) the lack of induction of clinical signs in the DSS colitis experiment, and (iii) the lack of mortality or evident clinical morbidity when the cages had the highest load of spores inside the isolators and during the monitoring period (housed inside gnotobiotic cages) in none of the 125 animals suggest that this pathogen has limited clinical effect on immunocompromised mice devoid of commensal flora.

Maintaining GF animals is an expensive and labor-intensive practice highly prone to undesirable contaminants. Although* Aspergillus* could be classified either as saprotrophic (decaying plant matter) or as clinical strains [7], the major risk for GF contamination is saprotrophic strains. The source of* A. sydowii* presence in our irradiated diet is uncertain, but it is possible that a batch of contaminated raw plant material (because* A. sydowii* is a saprophytic fungi of decaying plant matter that could be used for animal feed preparation) [9] served as source of spores that survived irradiation [18]. The amount of energy required to kill 90% (1 Log_10_) of the microbial population (LD10) for most* Aspergillus* spp. spores has been reported to be between 0.28 and 0.60 kGy [19]. Irradiation doses of 20–30 kGy are commonly used to treat diets intended for SPF animals, and doses of 40–50 kGy are recommended for diets intended for gnotobiotic or germ-free animals [20]. Although it was not possible to document the irradiation protocol used commercially by the supplier of feed to our GF colony, the resistance to irradiation is of bioengineering interest because it is increasingly known that dark pigmented fungi, via melanin molecules, seem to protect them in highly gamma-radiated environments, including outer space.* Aspergillus sydowii* is the third most common fungus in the NASA international space station [18]. With respect to pathogenicity in GF mice our findings cannot be extended to other* A. sydowii* strains because virulence seems to be strain-dependent based on studies characterizing clinical and environmental isolates [7]. Fungi (initially thought to be exclusively aerobic microorganisms) could however modify host-microbial responses in intestinal diseases due to their production of mycotoxins, especially if certain strains were capable of colonizing the anaerobic intestinal tract (e.g., as it commonly occurs in the forestomach of ruminants). In addition,* A. sydowii* could modify host-microbial responses in the gut in the presence of other commensal flora via epigenetic modifiers that alter the production of novel anti-inflammatory (sesquiterpenoid) molecules [21, 22]. If* Aspergillus *was capable of colonizing the anaerobic gut, it could modulate the microbiota and intestinal inflammation by the production of secretory molecules. This case report only highlights that the relevance of this and other fungi in IBD requires further studies to control for interactions with other elements important in the function of the digestive system, including the presence of various types of commensal and dysbiotic gut microbiota.
